# Assessing the Challenges in the Application of Potential Probiotic Lactic Acid Bacteria in the Large-Scale Fermentation of Spanish-Style Table Olives

**DOI:** 10.3389/fmicb.2017.00915

**Published:** 2017-05-17

**Authors:** Francisco Rodríguez-Gómez, Verónica Romero-Gil, Francisco N. Arroyo-López, Juan C. Roldán-Reyes, Rosa Torres-Gallardo, Joaquín Bautista-Gallego, Pedro García-García, Antonio Garrido-Fernández

**Affiliations:** ^1^Food Biotechnology Department, Instituto de la Grasa (Agencia Estatal Consejo Superior de Investigaciones Científicas, CSIC), University Campus Pablo de OlavideSeville, Spain; ^2^JOLCA Aceitunas S.A.Seville, Spain

**Keywords:** Green Spanish-style table olives, probiotic starter culture, large-scale fermentation, inoculum survival

## Abstract

This work studies the inoculation conditions for allowing the survival/predominance of a potential probiotic strain (*Lactobacillus pentosus* TOMC-LAB2) when used as a starter culture in large-scale fermentations of green Spanish-style olives. The study was performed in two successive seasons (2011/2012 and 2012/2013), using about 150 tons of olives. Inoculation immediately after brining (to prevent wild initial microbiota growth) followed by re-inoculation 24 h later (to improve competitiveness) was essential for inoculum predominance. Processing early in the season (September) showed a favorable effect on fermentation and strain predominance on olives (particularly when using acidified brines containing 25 L HCl/vessel) but caused the disappearance of the target strain from both brines and olives during the storage phase. On the contrary, processing in October slightly reduced the target strain predominance on olives (70–90%) but allowed longer survival. The type of inoculum used (laboratory vs. industry pre-adapted) never had significant effects. Thus, this investigation discloses key issues for the survival and predominance of starter cultures in large-scale industrial fermentations of green Spanish-style olives. Results can be of interest for producing probiotic table olives and open new research challenges on the causes of inoculum vanishing during the storage phase.

## Introduction

Table olives are a fermented vegetable of the Mediterranean basin with many centuries of history. Among the different types of olive elaborations, green Spanish-style is the most well-known industrial process (Garrido-Fernández et al., 1997). Borbolla y Alcalá et al. (1952) were among the first researchers to apply pure starter cultures in this elaboration. Since then, many investigations were conducted with the objective of ensuring the microbiological control of the fermentation process to produce adequate and safe products (Martorana et al., 2015; Tataridou and Kotzekidou, 2015). The adhesion of lactobacilli and bifidobacteria on the epidermis of the processed olives (Lavermicocca et al., 2005) and the formation of true biofilms on green Spanish-style table olives (Arroyo-López et al., 2012a; Domínguez-Manzano et al., 2012) have been reported recently. Such discoveries have encouraged researchers to investigate the use of multifunctional starter cultures for producing potential probiotic olives at laboratory and pilot plant scales. In fact, this step is an essential requirement to turn table olives into a carrier of beneficial microorganisms to the human body. Rodríguez-Gómez et al. (2013) assessed the technological characteristics and the dominance of several strains of *Lactobacillus pentosus* in 100 L fermentation vessels. Blana et al. (2014) used single and combined cultures of *L. pentosus* and *Lactobacillus plantarum*, isolated from industrially fermented olives, for processing Halkidiki olives in 14 L capacity plastic vessels. Both potential probiotic strains successfully colonized the olive surface, although *L. pentosus* B281 presented the most desirable characteristics for predominance. In heat shocked green olives, the results were similar, and higher recoveries of the *L. pentosus* B281 strain were observed (Argyri et al., 2014). The commercial probiotic *Lactobacillus rhamnosus* GG was also recovered from inoculated samples of natural green Italian cultivars, demonstrating its survival in the olive brine matrix (Randazzo et al., 2014). Sisto and Lavermicocca (2012) also reported the suitability of *Lactobacillus paracasei* IMPC2.1 to make olives with potential probiotic properties. Their results showed that the strain formed a biofilm on the fruits and persisted in high numbers during fermentation. The biofilm formation was also observed on Conservolea natural black olives processed with the functional starter culture *L. pentosus* B281 (alone or in co-inoculation with *Pichia membranifaciens*). Molecular analysis revealed that the bacteria successfully colonized the black olive surface and presented a high recovery rate. On the contrary, recovery of the yeast was limited (Grounta et al., 2016). Recently, Bevilacqua et al. (2015) made a review of the most relevant biotechnological innovations in table olives, based on both traditional and innovative starter cultures.

Despite the substantial effort devoted to the use of starter culture in green Spanish-style table olives, the fermentation process at industrial scale remains virtually spontaneous. Furthermore, the complete predominance of a starter culture at large-scale for producing fermented probiotic olives still constitutes an important technological challenge. The objective of this work was the investigation of the factors that may affect the survival/predominance of a multifunctional starter culture at a large-scale level (16 cubic meter fermentation vessels), under the current processing technology (open tops of containers and environmental conditions prevailing in the fermentation yards). Aiming at the selection of the most favorable conditions for the assurance of the appropriate strain implantation, the present study assessed the effects of time of inoculation, time of the processing season, inoculum history, and initial pH correction on the fermentation profile and inoculum predominance.

## Materials and Methods

### Experimental Design and Olives

This work is related to experiments carried out in two successive seasons: 2011/2012 (inoculation, 26 September 2011) and 2012/2013 (inoculations: 27 September 2012 and 25 October 2012). The study was carried out at JOLCA, S.A. facilities in Huevar del Aljarafe (Seville, Spain), using Manzanilla olives picked by hand at the green maturation stage.

The experiments were always carried out in 16,000 L industrial fermentation vessels, containing 9,856 kg olives and 6,374 L brine. The debittering process and washing were similar in both seasons, and the operations were always performed at environment temperature. The lye treatment was conducted in above-ground vessels of the olive debittering section of the industry, with a 2.6% NaOH solution, which was let to penetrate approximately 2/3 of the flesh (∼6 h). The lye was then removed, and tap water was added to cover the fruits. After 8 h washing, the exhausted water was substituted with an 11% (w/v) NaCl solution for initial salt absorption (∼10 h). Then, the olives were transferred into underground vessels situated in the fermentation yard. During this operation, the HCl proportions specified for each treatment (see below) were progressively incorporated to the circulating brine, to facilitate their homogeneous distribution.

**FIGURE 1:**
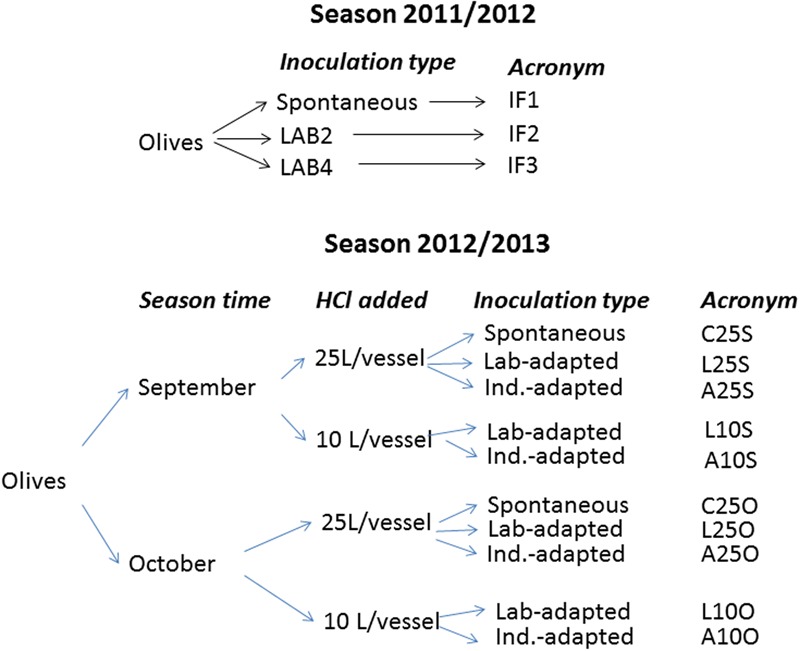
**Flow chart of the experimental design according to the season.** All experiments were performed in duplicate (independent fermentation vessels), and the HCl always was food grade quality (35%, w/w). The correspondence between variable levels and symbols are: September, S; October, O; 25 or 10 L HCl added in initial brine /vessel, 25 and 10, respectively; Spontaneous fermentation, C; culture pre-adapted at the laboratory or industrial scales, L and A, respectively.

In 2011/2012 season, the design consisted of three duplicate independent treatments (in total six fermentation vessels) which were identified as IF1 (control, spontaneous), IF2 (inoculated with TOMC-LAB2, LAB2 in short) and IF3 (inoculated with TOMC-LAB4, LAB4 in short) (**Figure 1**). The LAB strains were selected because of their promising potential probiotic characteristics (Bautista-Gallego et al., 2013; Arroyo-López et al., 2014). In 2011/2012 season, all fermentation vessels were filled with the same acidified brine [25 L food grade HCl (35% w/w)/vessel]. After allowing 3 days for partial equilibrium, the inoculation was performed by adding 5 L of laboratory-adapted *L. pentosus* LAB2 and LAB4 cultures. The pre-adaptation was achieved by growing the two strains in MRS broth (Oxoid LTD, Basingstoke, England), added with 4% NaCl, till the stationary phase was reached (24 h).

In 2012/2013 season, the design included the following variables: processing time (September vs. October), inoculum type (laboratory vs. industry pre-adaptation), and the proportion of acid added to the initial brine [25 vs. 10 L food grade HCl (35%, w/w)/vessel]. Therefore, for each season time, the experiment consisted of a duplicate complete 2^2^ factorial design on inoculum type and proportion of acid added plus the corresponding controls without inoculation. The design required a total of 10 industrial fermentation vessels. The temperature averages during processing were 27 and 22°C in September and October, respectively. The acronyms of the treatments were obtained by combining the variable (history/type of inoculation, HCl volume added, and processing time) levels. E.g., A25S, refers to a treatment performed in September (S), using a brine added with 25 L HCl (35% w/w), and inoculated with a pre-adapted in the industry starter (A). A flow chart with a detailed explanation of the experimental designs and the acronyms of treatments is shown in **Figure 1**. In 2012/2013, based on the results of the previous season, only the *L. pentosus* LAB2 strain was used, and the inocula were added twice (when the olives were transferred into the underground fermentation vessel and at the following day).

### Bacterial Strains and Brine Inoculation

In season 2011/2012, cultures (LAB2 and LAB4) were prepared and pre-adapted in the laboratory using 5 L flasks with MRS broth supplemented with 4% NaCl and 2.5% glucose. When the populations reached the stationary phase, approximately 9 log_10_ CFU/mL according to plate counting in MRS agar medium, the contents of the flasks were introduced directly into the fermentation vessels. The inoculum was distributed into the large fermentation vessels by using a pump provided with an appropriate sterile stainless steel tube, which was introduced at diverse deeps and directions. Later, the fermentation brines were fully homogenized, without aeration, by recycling the solution with a pump.

In 2012/2013 season, the laboratory pre-adapted inoculum (L) was prepared similarly to those of the 2011/2012 season but using LAB2 only. For the preparation of the industry pre-adapted to brine inoculum (A), 1 L of a previously grown in MRS LAB2 culture was added to a plastic drum, containing 200 L of fresh brine, which had the following composition: 4% NaCl, 0.1% MRS broth and 2.5% glucose. The drums were let at environment temperature (20–30°C) for 1 day, after which the LAB2 population reached ∼8 log_10_ CFU/mL. Then, 100 L of the pre-adapted inoculum were added to 10, and 25 L HCL treated fermentation vessels, using the same procedure of 2011/2012 season. The operation (re-inoculation) was repeated at the following day (24 h) to counteract the usual partial initial population decay after the first inoculum addition. Therefore, each fermentation vessel was inoculated twice.

### Physicochemical Analysis

The analyses of pH, titratable and combined acidity in the cover brines were performed in samples withdrawn from the bottom, half and top of the fermentation vessels, using standard methods (Garrido-Fernández et al., 1997). Their averages were recorded and used for further studies.

### Microbiological Analysis

The brine samples (100 μL), withdrawn at selected fermentation periods, were plated [directly or after appropriate dilutions with a sterile peptone water (0.9% peptone, wt/vol)], using a Spiral System model dwScientific (Dow Whitley Scientific Limited, England) on specific selective media. The use of an automatized spiral system reduces considerably the error associated with the plating methodology. *Enterobacteriaceae* were counted on Crystal-violet Neutral-Red bile glucose (VRBD) agar (Merck, Darmstadt, Germany), lactic acid bacteria (LAB) were spread onto de Man, Rogosa and Sharpe (MRS) agar (Oxoid) supplemented with 0.02% (wt/vol) sodium azide (Sigma, St. Louis, MO, USA), and yeasts were grown on yeast–malt–peptone–glucose medium (YM) agar (Difco, Becton and Dickinson Company, Sparks, MD, USA) supplemented with oxytetracycline and gentamicin sulfate (0.005%, wt/vol) as selective agents. The plates were incubated at 30°C for 48 h (bacteria) and 72 h (yeasts) and counted using a Flash&Go (IUL, Barcelona, Spain) image analysis system. Brine counts were expressed as log_10_ CFU/mL.

To determine the microorganisms adhered to the olive epidermis, the enzymatic protocol developed by Böckelmann et al. (2003) for the detachment of biofilms was followed. Briefly, fruits (25 g) from each fermentation vessel were randomly taken and washed for 1 h with 250 mL of a sterile phosphate buffered saline (PBS) solution. Then, the olives were transferred into 50 mL of a PBS solution with the following enzymes: 14.8 mg/L lipase (L3126), 12.8 mg/L β-galactosidase (G-5160), and 21 μL/L α-glucosidase (G-0660) (Sigma-Aldrich, St. Louis, MO, USA). To achieve biofilm disintegration and removal of the adhered cells, the fruits were incubated at 30°C in this enzyme cocktail with slight shaking (150 rpm). After 12 h, the olives were removed, and the resulting suspension centrifuged at 9,000 × *g* for 10 min at 4°C. Finally, the pellet was re-suspended in 2 mL of PBS and spread onto the different culture media described above. Olive counts were expressed as log_10_ CFU/g olive (average weight and surface: 4.08 ± 0.46 g and 10.99 ± 1.01 cm^2^, respectively; *n* = 50).

### Characterization of LAB Populations Adhered on Olives Epidermis

For characterization of the lactobacilli population, repetitive bacterial DNA element fingerprinting analysis (rep-PCR) with primer GTG_5_ was followed using the protocol described in Versalovic et al. (1994). The PCR reaction in a final volume of 25 μL contained: 5 μL of 5x MyTaq reaction buffer (5 mM dNTPs and 15 mM MgCl_2_), 0.1 μL of My Taq DNA polymerase (BiolineReactives, United Kingdom), 1 μL GTG_5_ primer (25 μM), 13.9 μL deionized H20 and 5 μL DNA (∼20 ng/μL). PCR amplification was carried out in a thermal cycler (Master Cycler Pro, Eppendorf) with the following program: 95°C for 5 min as initial step plus 30 cycles at: (1) denaturation at 95°C for 30 s, (2) annealing at 40°C for 1 min, and (3) extension at 65°C for 8 min, with a final step of 65°C for 16 min to conclude the amplification. This methodology was used to determine the recovery frequency of the inoculated strain at the time of the maximal population of LAB. Ten isolates were randomly picked from each fermentation vessel, making a total of 160 isolates for the two seasons. Their pattern profiles of bands (from 100 up to 3,000 bp) were compared with the strains used to inoculate the fermentation vessels (LAB2 and LAB4). For this purpose, PCR products were electrophoresed on a 2% agarose gel and visualized under ultraviolet light by staining with ethidium bromide. The resulting fingerprints were digitally captured and analyzed with the BioNumerics 6.6 software package (Applied Maths, Kortrijk, Belgium). The similarity among digitalized profiles was calculated using the Pearson product-moment correlation coefficient. Dendrograms were obtained by means of the Unweighted Pair Group Method using Arithmetic Average (UPGMA) clustering algorithm and the automatic calibration tool for the determination of the optimization and curve smoothing parameters.

### Scanning Electron Microscopy

For “*in situ*” observations of the microbiota adhered to the olive epidermis, the method developed by Kubota et al. (2008) was followed. Olives were taken from each fermentation vessel at the moment of maximum population of LAB and washed twice for 1 h with a 100 mM phosphate buffer (pH 7.0). Then, the fruits were placed for 2 h in the same phosphate buffer with 5% glutaraldehyde and then washed several times. Slices (0.5 cm^2^) of the olive epidermis were dehydrated in increasing concentrations of ethanol (50, 70, 80, 90, 95, and 100%) and fixed onto glass slides. Finally, samples were sputtered with gold, using a Scancoat Six scanning electron microscopy (SEM) sputter coater equipment (Edwards, Gat, Israel), for 180 s and observed with a scanning electron microscopy model JSM-6460LV (Jeol, Ltd, Tokyo, Japan).

### Data Analysis

The physicochemical and microbiological characteristics of each treatment were followed, and their averages recorded. The effect of the treatments was studied by following throughout processing the averages (*n* = 4) of each variable level (l = 2) over the two other variables. Simultaneously, their confidence intervals (*p* = 0.05) at each sample point were also estimated. As an approximation, the observation of non-overlapping confidence intervals of the two levels, at several successive sampling points, was considered as a significant effect of the variable in that interval; that is, during that time, the analyzed variable exerted a significant effect (difference) on such parameter at *p* < 0.05. The typical estimations of the effects over time were not intended because not all the fermentation vessels were sampled at the same time and the measures over time were not considered as independent. When necessary, the scales were re-adjusted to facilitate comparisons among treatments. The data analyses and graphs were performed using SigmaPlot release 13.0 (Systat Software, Inc., 2008).

## Results and Discussion

The survival/predominance of starter cultures at large-scale fermentations, in the current state of the art of the industrial technology, is not a trivial issue. Ruiz-Barba and Jiménez-Díaz (2012) have presented a novel *L. pentosus* paired starter culture that was tested in several industrial 10-ton vessels. Apparently, the starter rapidly colonized the brines, dominated the native microbiota, and persisted throughout fermentation. However, this is not usual. De Bellis et al. (2010) inoculated industrial plastic vessels (190 kg olives) with the probiotic strain *L. paracasei* IMPC2.1, using 4 and 8% NaCl at room temperature and 4°C. Results indicated that the *L. paracasei* strain successfully colonized the olive surface and dominated the LAB population. Besides this, there was a considerable genetic diversity of LAB species colonizing the olive surface, mainly at 8% NaCl. Lucena-Padrós et al. (2014) have confirmed this microbial diversity at industrial scale in a recent survey carried out at several locations; the spontaneous fermentation was dominated by *L. pentosus*, but there was always a diverse secondary microbiota, including some new bacterial species. In fact, the actual processing conditions prevailing in the current fermentation yards together with the industrial high volume vessels with considerable brine surface exposed to air makes difficult the survival of any starter culture over the environmental microbiota by just controlling the physicochemical conditions. If the objective is the control of the microbiota adhered to the olive surface, the challenge may be even harder. Therefore, establishing conditions for starter culture survival/predominance not only in the cover brine but also on the olive biofilms is a priority for the production of potentially probiotic olives. The present work was designed with this objective.

Among other environmental factors prevailing during green Spanish-style fermentation, several studies consider salt concentration as playing an important role for LAB inoculum survival (De Bellis et al., 2010; Blana et al., 2014). However, in this work, it was let constant (∼6%, w/v) at the equilibrium due to the vast acceptance of this level by industry (Garrido-Fernández et al., 1997). Furthermore, recent studies have demonstrated that LAB growth is usually inhibited at higher than 8% salt concentrations (Romero-Gil et al., 2013). Also, some starter cultures can be applied in alkaline conditions (Sánchez et al., 2001) or without pH correction (Ruiz-Barba and Jiménez-Díaz, 2012), although the addition of diverse proportions of HCl in the initial brine is rather common. In fact, its incorporation reduces the exposure of fermenting olives to high pH levels and limits the riks of undesirable microbial populations such as *Enterobacteriaceae* (Garrido-Fernández et al., 1997). Hence, acidification of initial brines was adopted, although with different levels according to treatments. The use of these conditions was forced by industry to prevent any risk that might compromise the quality and safety of the product involved in the experiment (16 fermentation tanks of 10-ton olives, for a total of about 157.696 kg of fruits).

### Tentative Inoculation (2011/2012 Season)

The results obtained in 2011/2012 season constituted the basis for the selection of the treatments applied in the following trial of experiments (2012/2013 season). Thus, they merit some concise comments. The main interest of this tentative essay was focussed on the performance/predominance of the LAB strains, using inoculation conditions based on the literature. In general, the information on this aspect is limited. De Bellis et al. (2010), Ruiz-Barba and Jiménez-Díaz (2012), and Blana et al. (2014) give detailed information on the experiments but not on when the inocula were added. Therefore, the inoculation of microorganisms was performed on the third post-brining day when, supposedly, the solution was already transformed into a sufficiently nutritious fermentation medium. In further experiments on the same fermentation type, Rodríguez-Gómez et al. (2013) applied a similar post-brining delay before inoculation.

In general, the changes in the physicochemical characteristics during fermentation followed the typical trend for green Spanish-style table olives (Garrido-Fernández et al., 1997). It was characterized by a fast pH decrease, a simultaneous (although of opposed direction) and rapid production of acid (which took place in a reduced period, ∼20 days), and a fast combined acidity and salt concentration equilibrium. In brine, the microbial population was composed of *Enterobacteriaceae* (for a short period), LAB and yeasts. On olives, the images showed the typical biofilm formation (Arroyo-López et al., 2012a; Rodríguez-Gómez et al., 2013), even in the spontaneous fermentation (control). The biofilms were mainly composed of LAB and yeast, whose counts indicated a high population at the ∼20th day which, as usual, slowly declined after the fermentation phase (Rodríguez-Gómez et al., 2013). However, unexpectedly, the biofilm also included *Enterobacteriaceae*, which was still detected after 3 months of fermentation (a longer period than in brine), but not at the end of the process (population below the detection limit, 1.2 log_10_ CFU/mL). Apparently, their embedding in the biofilm might have supported a longer presence on the olives than in the brine. In any case, the LAB control on the biofilms showed that from the 15th day (possible the moment of the maximum population) onward, the profiles of the isolates from the spontaneous fermentation (control) had low similarities with those from the inocula. Please, consult the Supplementary Figures S1–S3 to obtain more information of these processes.

Therefore, if only the standard brine control had been followed, the fermentation with LAB2 and LAB4 would have been assessed as successful; but, in fact, it failed since the objective of the inoculum predominance was not achieved. However, the experiment was useful for showing that the industrial probiotic fermentations require strengthening the hygienic conditions (for preventing the occurrence of *Enterobacteriaceae* in the biofilms) and re-considering the conditions of inoculation. A way of improving starter survival/predominance could be the correction of the initial pH by HCl addition and the incorporation of the LAB inoculum as soon as possible, which could serve as a mechanism of biocontrol and promotion of the proper fermentation. The experiments of the following season were performed considering these aspects.

### Effects of the Variables Studied in the Second Season (2012/2013) on the Fermentation Profile

Based on the previous results, in this season the starter was always added just after transferring the olives into the underground fermentation vessels (to reduce as much as possible the initial wild population, including *Enterobacteriaceae* and spontaneous LAB). The variables studied in this season, were: (i) initial pH of the brine (controlled at two levels by 25 L vs. 10 L HCl addition), (ii) processing time [at the beginning of the season (September), olives are richer in polyphenols and sugars, and the temperature is more favorable than in October], and (iii) inoculum history (laboratory vs. industry pre-adaptation, which might facilitate its rapid growth in the industrial vessels).

**FIGURE 2:**
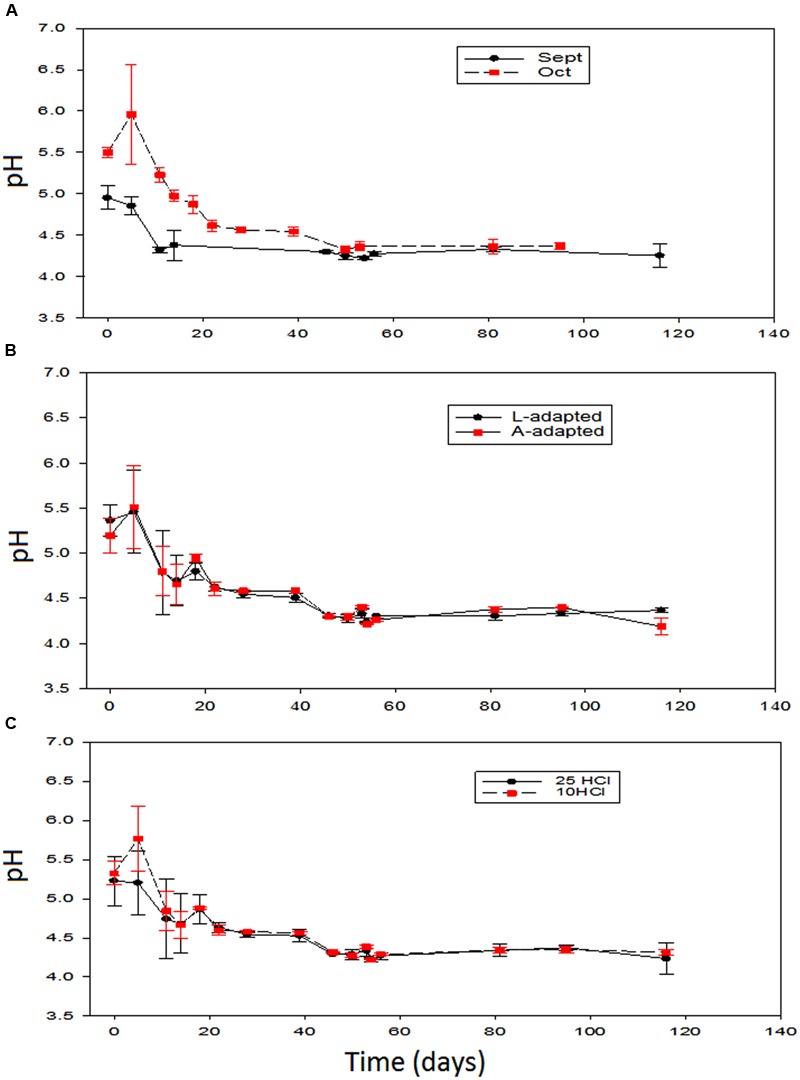
**Application of potential probiotic starter (LAB2) at industrial scale (season 2012/2013).** Changes in pH (± CL, *n* = 4 for each level) according to time season **(A)**, type of inoculum **(B)**, and proportion of HCl added to fermentation vessels **(C)**. The values are always averages over the other two non-included in the graph variables. See **Figure 1** for the the meanings of symbols.

It is noteworthy that the average initial pH in the just filled fermentation vessels were 5.1 (± 0.2), in September (beginning of the season), and 5.5 (± 0.1), in October (final of the season). The effects of the different variables on this parameter had been stressing. Only the season time showed a significant effect during the first 40 days (**Figure 2**). The pH level was initially higher in the olives processed in October than in those prepared in September, although the differences were progressively decreasing and disappeared at about the 50th day. After this time, the evolution was similar. Notice that the slight increase in pH in October, due to the leaching of the NaOH excess before reaching equilibrium, could, eventually, have been stressing for LAB growth (Sánchez et al., 2001). In the treatments processed in September, the pH decreased quite rapidly during fermentation and reached the equilibrium at around the 18th day after brining. However, in those prepared in October, after the first increase, the pH decrease was slower, and the equilibrium was reached later, at around the 50th day due to the lowest LAB counts during the initial fermentation phase (see below). On the contrary, the changes in pH over the other variables showed that the type of inoculum and the proportion of HCl added to the initial brine had not significant effects on this parameter (**Figures 2B,C**). In general, the changes in pH were similar to those observed in other experiments with Greek cultivars (Blana et al., 2014) or to those observed inoculating *L. paracasei* IMPC2.1, a strain of human origin, in Bella di Cerignola debittered green olives (Sisto and Lavermicocca, 2012).

**FIGURE 3:**
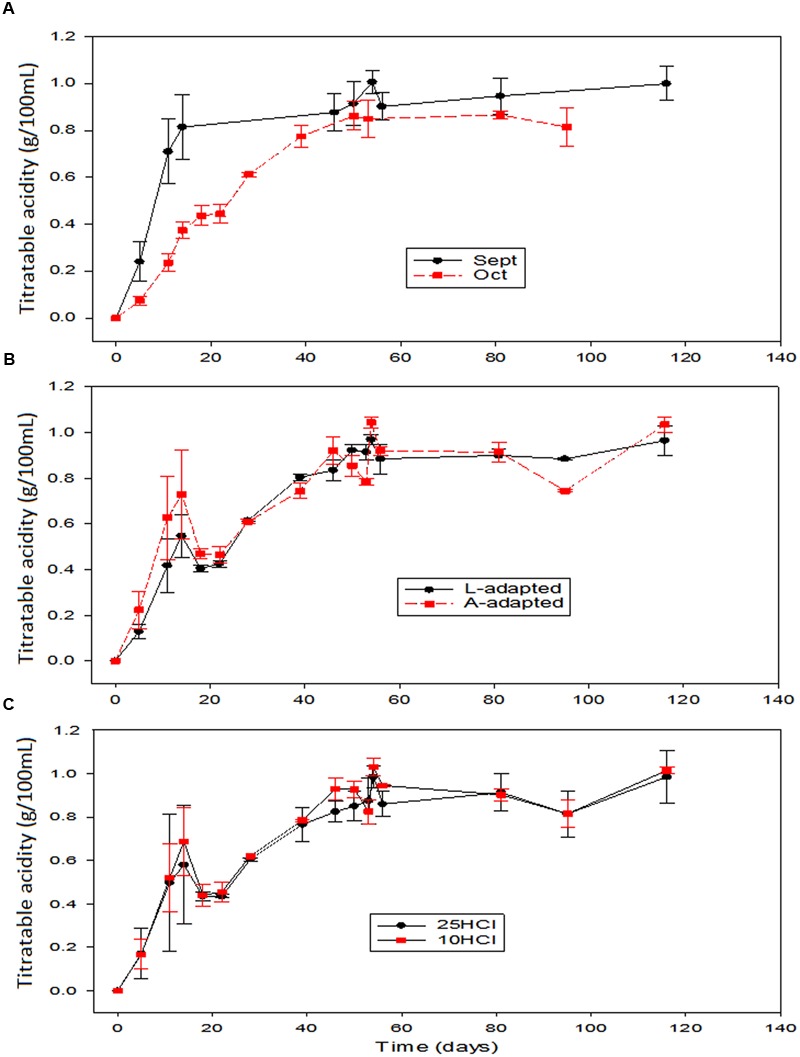
**Application of potential probiotic starter (LAB2) at industrial scale (season 2012/2013).** Changes in titratable acidity (± CL, *n* = 4 for each level) according to time season **(A)**, type of inoculum **(B)**, and proportion of HCl added to fermentation vessels **(C)**. The values are always averages over the other two non-included in the graph variables. See **Figure 1** for the the meanings of symbols.

The titratable acidity over time was influenced again by the season time (**Figures 3A**), being the acid formation faster in September when most of the acid was produced before the 20th fermentation day. The onward production was rather slow, with concentrations showing a progressive increase. The average titratable acidity in the spontaneous fermentation corresponding to this time (data not shown) was slightly above those of the other fermentation vessels during storage, possibly due to the LAB disappearance in the inoculated vessels after fermentation. In the treatments applied in October, the titratable acidity was formed more progressively (lower production rates than in September) but following a similar trend in all fermentation systems (even control) (**Figure 3A**). Hence, the performance of *L. pentosus* LAB2 at industrial scale behaved a little bit worse than the wild LAB populations in September but similarly to them in October. Hence, the effect of processing time on titratable acidity (**Figure 3A**) showed that, in September, the acid production rates (and their levels) were clearly above those treated in October not only during the 50 first days but also in the storage phase. Furthermore, the titratable acidity in the olives processed in September did not decline during the study while tended to decrease along the storage in the vessels processed in October, probably due to a certain progressive consumption of the lactic acid by yeasts (Garrido-Fernández et al., 1997). However, the changes in the titratable acidity were not affected by the type/history of inoculation used or by the proportion of HCl added to the initial brine (**Figures 3B,C**). During large-scale green Spanish-style table olive fermentation devoted to probiotic production using Halkidiki cultivar, a faster acidification at 8% NaCl than at 10% was observed (Blana et al., 2014). In the present study, the salt levels in the equilibrium were initially in the range 6–8%; then, the slowest acid production in October vessels may have been due to the effect of a low sugar concentration at this time (ripening of fruits) or to the level of temperature, about 5°C lower in October.

With respect to other physicochemical characteristics, the initial salt concentration was 6.7–7.6°Bé in September, and 6.4–6.7°Bé in October. However, such difference hardly affected LAB growth since it would eventually had favored them in in the olives processed in October. In any case, since no specific control was exerted on this parameter, their evolutions should be considered as the habitual for this processing at the season times essayed (Garrido-Fernández et al., 1997). The combined acidity plays an important role during fermentation, due to its buffering capacity (Garrido-Fernández et al., 1997). The combined acidity levels in brine rapidly rose due to the organic matter leakage into the brine. No effect of season time, type of inoculation, and HCl added to the initial brines were observed on salt or combined acidity, although fermentation vessels with 25 L HCl added led, as expected, to the lowest non-significant levels of the last parameter. More information on the evolution of these parameters during fermentation are included in the Supplementary Figures S4, S5.

Regarding microbial populations, the *Enterobacteriaceae* were detected only in brine during the first fermentation week and always below 3 log_10_ CFU/mL (data not shown), confirming that the initial pH correction had a favorable effect on the control of these microorganisms (Garrido-Fernández et al., 1997). This behavior contrast with the *Enterobacteriaceae* presence during about the 30 days period reported by De Bellis et al. (2010) in experiments inoculated with *L. paracasei* IMPC2.1. As expected, the LAB population was higher in the inoculated fermentation brines than in the control fermentation vessels (∼6.0 vs.∼ 2.0–3.2 log_10_ CFU/mL), regardless of treatments. In September, such difference was maintained during all the phase of active fermentation. On the contrary, in October, the initial LAB populations in several treatments decreased, but the decay was followed by later recoveries (data not shown).

**FIGURE 4:**
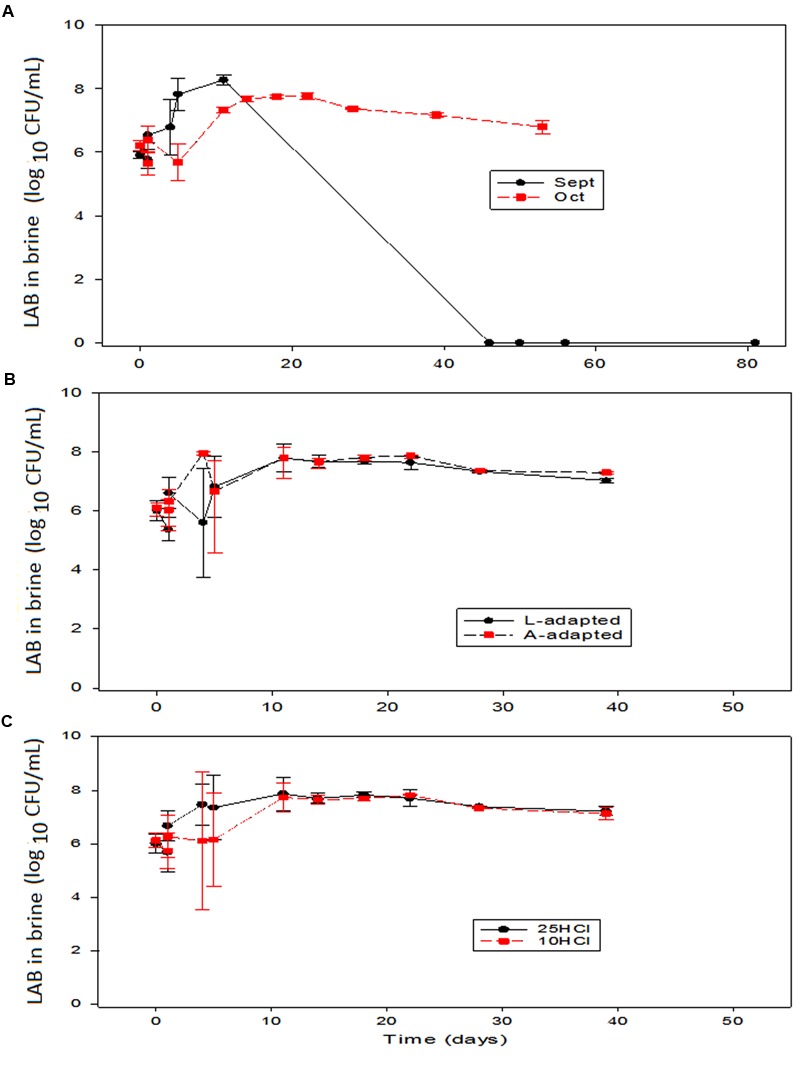
**Application of potential probiotic starter (LAB2) at industrial scale (season 2012/2013).** Changes in LAB in brine (± CL, *n* = 4 for each level) according to time season **(A)**, type of inoculum **(B)**, and proportion of HCl added to fermentation vessels **(C)**. The values are always averages over the other two non-included in the graph variables. See **Figure 1** for the meanings of symbols.

The trends followed by LAB were quite dependent on the processing time (**Figure 4**). In general, the populations in the vessels inoculated with starter cultures followed entirely different trends along the first fermentation period, which was characterized by an immediate growth in September vs. slight decrease followed by a recovering in October (**Figure 4A**). This different behavior resulted in September in a faster rapid growth of the LAB, that reached a maximum average population slightly above those treatments processed in October. The differences between the LAB in brine behaviors according to processing times were also observed along the fermentation. In September, the LAB populations decreased sharply and disappeared around the 40th day. In the treatments processed in October, on the contrary, the LAB population only suffered a slight progressive decrease after the maximum (**Figure 4A**). Apparently, there were some environmental characteristics in September inoculated experiments that strongly, and preferably, affected LAB2 and caused its disappearance after fermentation. Among them, olive polyphenols (with higher concentrations in September than in October, Medina et al., 2010) or the own LAB metabolites (lactic acid or hydrogen peroxide) produced during fermentation. However, in September, such characteristics affected less markedly the wild LAB population (control) which survived at a moderate level (4 log_10_ CFU/mL) (data not shown). Conversely, the strong inhibitory effect above-commented was not observed in October, when the LAB population after fermentation always followed a trend similar to that found in normal processes (Panagou et al., 2003). The effects of the type of inoculation or the amount of HCl in the initial brines on the LAB populations in brine over the other two variables were never significant (**Figures 4B,C**). Argyri et al. (2014), on the contrary, did not find any sharp LAB decrease during fermentation or storage, indicating that their potential probiotic *Lactobacillus* were more resistant to inhibitors, the heat shock could have transformed them, or the strain did not produce toxic substances. Furthermore, in the case of the inoculation with *L. paracasei* IMPC2.1 Italian green olives, the LAB population also had a noticeable important decrease (De Bellis et al., 2010). In the case of the olive inoculation with *L. paracasei* IMPC2.1, the inoculum reduced the population of wild *L. pentosus* during the fermentation process but did not prevent its predominance from the 20th day up to the end of the process (Sisto and Lavermicocca, 2012).

**FIGURE 5:**
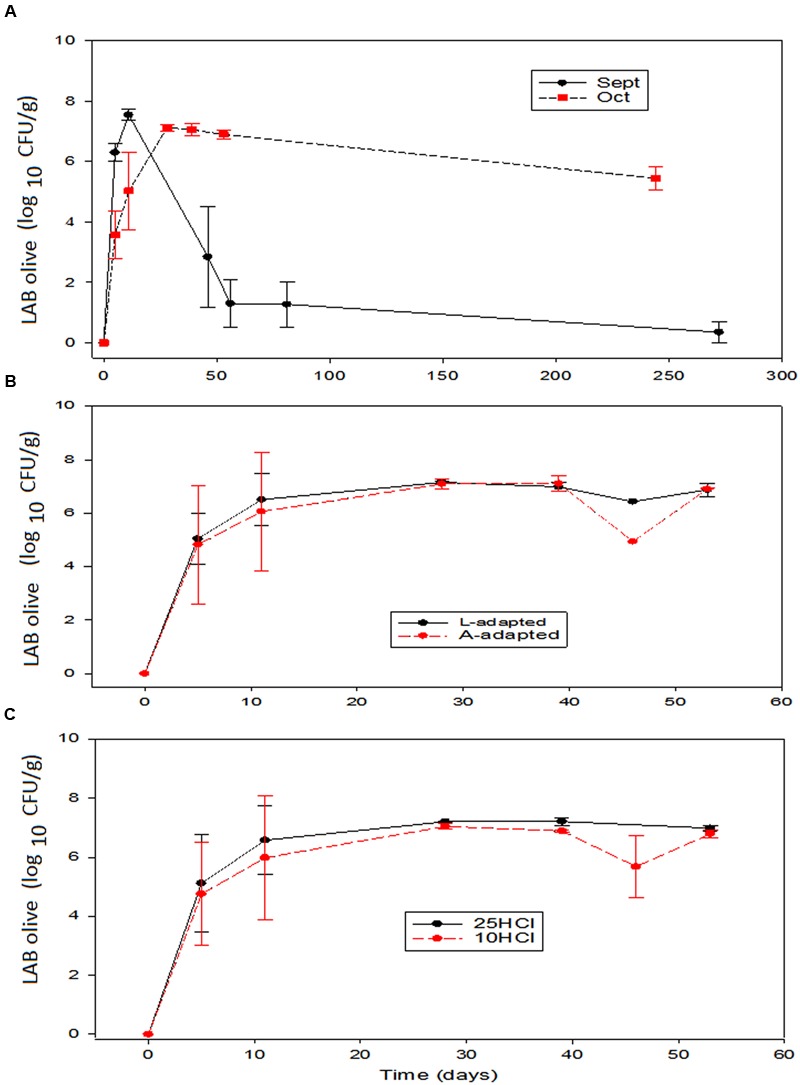
**Application of potential probiotic starter (LAB2) at industrial scale (season 2012/2013).** Changes in LAB on olives (± CL, *n* = 4 for each level) according to time season **(A)**, type of inoculum **(B)**, and proportion of HCl added to fermentation vessels **(C)**. The values are always averages over the other two non-included in the graph variables. See **Figure 1** for the meanings of symbols.

The presence of biofilm on the olives was detected shortly after brining and only included LAB and yeasts. Then, the composition was similar to that already found at pilot plant scale (Rodríguez-Gómez et al., 2014a). LAB population changes were rather similar to those observed in brine, and the main effect on olives was that of processing time (**Figure 5A**). Inoculation caused a more rapid biofilm formation and a slightly higher maximum population in September. However, after reaching the maximum, the trends followed depended on the processing time. The LAB counts on olives processed in September had a similar rapid decline than in brine and this population rapidly reduced below the detection limits (≤1.2 log_10_ CFU/mL). Therefore, the same inhibitory effect on LAB observed in September processed brines was also noticed on the LAB adhered to the olive surface. The causes of such inhibitions are probably the same above-mentioned for brines (Medina et al., 2010). On the contrary, the LAB population on the olives processed in October had only a slight decline in the storage period, and the survival was extended for above 250 days (**Figure 5A**). The survival on the fruits processed in October was then comparable to that found on the olives preserved in bulk, where a negative effect of the salt is always observed (Rodríguez-Gómez et al., 2014b). Conversely, the same organisms were not inhibited in similar experiments carried out at pilot plant scale (Rodríguez-Gómez et al., 2014a). Finally, inoculation type and the amount of HCl added caused negligible effects on LAB growth and survival (**Figures 5B,C**).

Regarding the evolution of yeast populations, it was observed an initial decreasing trend in brine, followed by an increase during the first period of fermentation to reach population levels around 4 log_10_ CFU/mL. Then, their counts did not show significant changes. For a period (between the 18th to the 40th day), the use of industry- pre-adapted starter culture, apparently, led to a lower yeast population than the laboratory-adapted one. The effect of the proportion of HCl added to the initial brine on the yeast populations was never significant. In general, yeasts counts were always comparable to those habitually found in green Spanish-style table olives during fermentation (Garrido-Fernández et al., 1997; Arroyo-López et al., 2012b). Furthermore, their levels were also comparable with those reported in other probiotic fermentation experiments in which the yeast growth was also allowed spontaneous (De Bellis et al., 2010; Argyri et al., 2014). In the case of the growth of yeasts on the olive surface, the yeast populations on the olives processed in September were generally above those found on fruits processed in October. On the contrary, no appreciable effects of inoculation and proportion of HCl added to the initial brine on yeast population on olives were observed. However, yeasts were found on the olive biofilm during the entire fermentation and storage period, in agreement with the role attributed to yeast on the initiation of the biofilm formation (León-Romero et al., 2016). Their changes followed similar trends to those found in other investigations (Argyri et al., 2014; Rodríguez-Gómez et al., 2014a). Supplementary Figures S6, S7 give more information of the evolution of this microbial group.

### Predominance of the Inoculum on the Olive Surface in the Second Season (2012/2013)

A molecular genotyping was performed to all the LAB isolates obtained from the biofilms at the moment of the maximum population to determine the frequency of recovery of the inoculum in the diverse treatments assayed.

**FIGURE 6:**
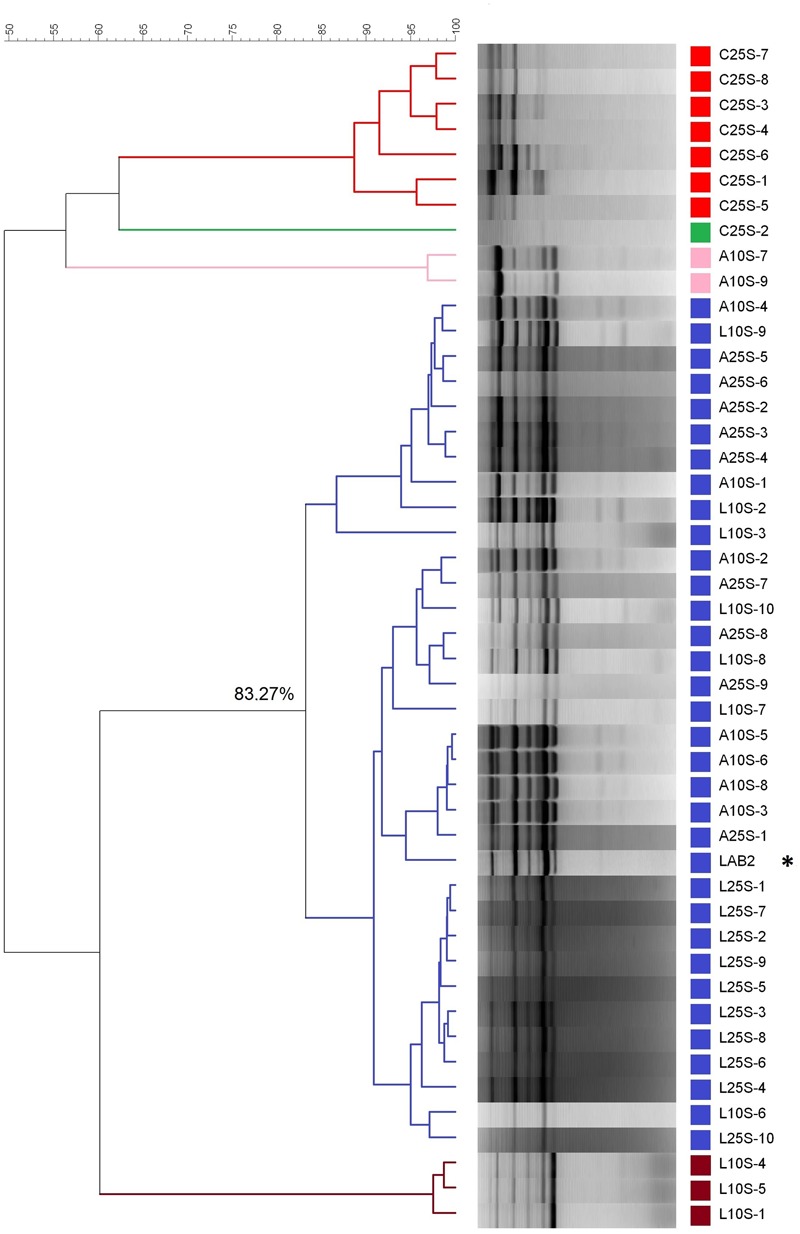
**Clustering analysis of the diverse LAB isolates obtained from the fermentation experiments carried out in September during 2012/2013 industrial fermentations.** L and A stand for laboratory and industry pre-adapted inocula, respectively; 25 and 10 stand for the proportions of HCl/vessel added to the initial brine; C stands for the spontaneous fermentation. The last number refers to the order of isolation. ^∗^Stands for the profile of inoculum LAB2.

**FIGURE 7:**
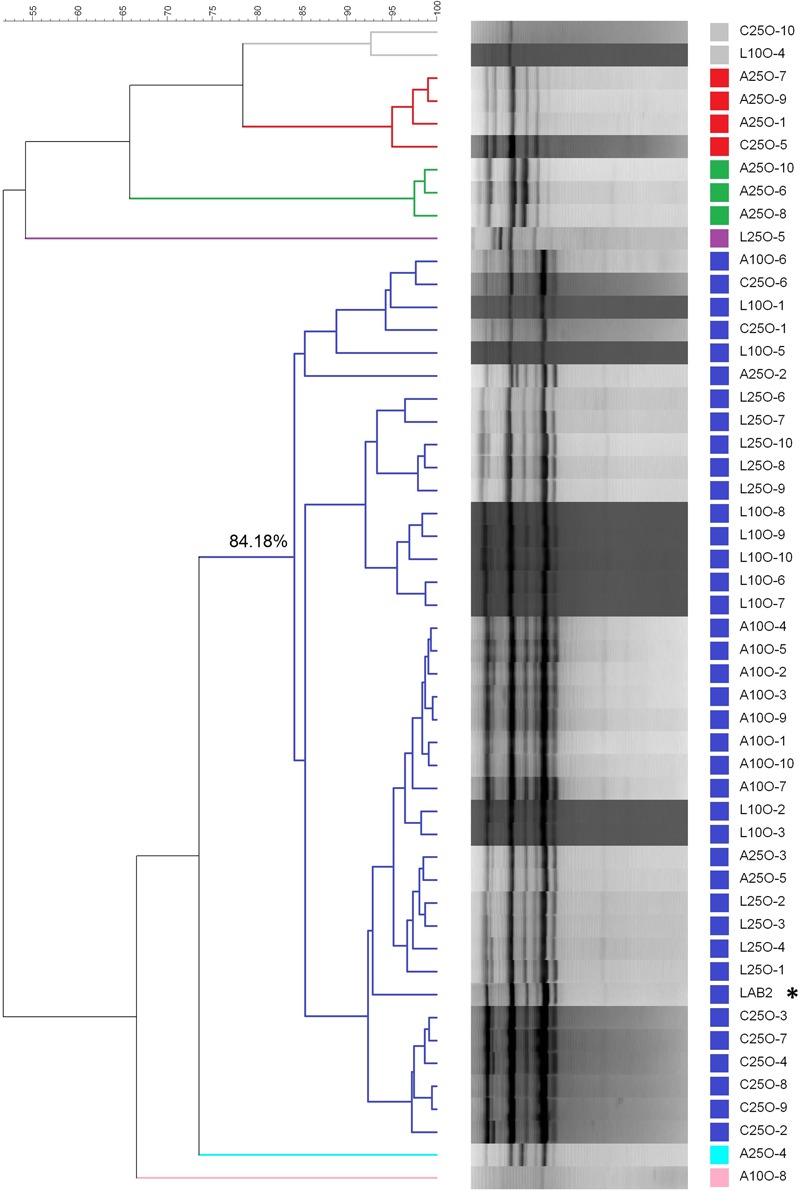
**Clustering analysis of the diverse LAB isolates obtained from the industrial fermentation experiments carried out in October during 2012/2013 season.** L and A stand for laboratory and industry scale pre-adapted inocula, respectively; 25 and 10 stand for the proportions of HCl/vessel added to the initial brine; C stands for the spontaneous fermentation. The last number refers to the order of isolation. ^∗^Stands for the profile of inoculum LAB2.

The dendrogram, based on the rep-PCR profiles using the GTG_5_ primer, revealed the presence of five clusters (below 83% similarity) in the isolates from olives processed in September (**Figure 6**). This cut-point was chosen according to previous studies carried out by Rodríguez-Gómez et al. (2014c), who found a reproducibility of 86.9 ± 3.4% in assays with LAB2 inoculum. On the one side, the isolates from the control treatment (C25S), spontaneous fermentation, were clearly different from the rest of the strains, with which, in the best case, only shared a 60% similarity. The biggest cluster was composed of 33 isolates, which included the LAB2 strain making the 71.73% of the total of isolates obtained. The percentage similarity shared within them was above 83%. Therefore, all these isolates could be assimilated to the inoculum profile. The other three groups consisted of one, two and three isolates respectively and shared a low similarity with the LAB2 inoculum. With respect to the treatments processed in October, the molecular analysis was carried out on 50 isolates (10 for each treatment). The dendrogram showed the presence of seven clusters below 83% similarity, some of them with only one isolate (**Figure 7**). The most numerous consisted of 38 isolates and had a minimum 84% similarity with the LAB2 profile, making 76% of total isolates obtained. Also most of the isolates from the spontaneous control (C25O) were included in this cluster, even if apart from those isolates from the olives of the inoculated fermentation vessels, possibly due to a process of cross contamination of this fermentation vessel. The other groups included the rest of the isolates, which shared a relatively low proportion of similarly. These isolates could hardly be related to the profile of the inoculum because the maximum similarity observed with it was 75% and most of them were even below 55%. Similarities of LAB2 in the case of this work were slightly higher than those observed at pilot scale (79%) (Rodríguez-Gómez et al., 2014a).

Based on the dendrograms from the LAB on olives in treatments processed in September and in October, the predominance (%) on the particular treatments were inferred (**Table 1**). The results showed that the predominance was not dependent on the type of inoculum used (laboratory or industry-adapted) but was always higher in September than in October. Also, the use of 25 L HCl/fermentation vessels for the pH correction led to fairly good predominance in September (100% in L25S and A25S) but slightly lower in October (90.3%). The results showed that, despite the current fermentation yard drawbacks, a 100% predominance of the inoculated strains could be possible, using a strong strain starter culture like *L. pentosus* LAB2 with proved ability for predominance at laboratory and pilot plant scales (Rodríguez-Gómez et al., 2014a).

**Table 1:** Predominance (expressed as percentage) of the inoculated *Lactobacillus pentosus* LAB2 strain at the moment of maximum LAB population in the diverse treatments assayed.

	Treatments applied in September (S)					Treatments applied in October (O)				
	A25S	A10S	L25S	L10S	C25S	A25O	A10O	L25O	L10O	C25O
Inoculum	100	78	100	70	0	30	90	90	90	80
^∗^Other LAB	0	22	0	30	100	70	10	10	10	20

*^∗^Percentage (%) of the predominance of other spontaneous LAB profiles. A stands for pre-adapted inoculum in the industry, while L stands for inoculum adapted in the laboratory. 25 stands for the addition of 25 L of HCl to fermentation tanks, while 10 stands for the addition of 10 L. C stands for the control treatments.*

Therefore, *L. pentosus*, in general, is a very well-adapted species to the green Spanish-style fermentation processes. In fact, Tofalo et al. (2014) found that *L. pentosus* was the dominant species (53–69%) in diverse Italian table olive fermentations. Blana et al. (2014) reported predominance frequencies ranging from 80 to 100% of their *L. pentosus* starter in Halkidiki fermentations. Argyri et al. (2014) also indicated in heat shocked olives high recoveries (100 or 95%, in 10 and 8% NaCl brines, respectively) for *L. pentosus* B281 but markedly lower proportions (59 or 55, in 10 and 8% NaCl brines, respectively) for *L. plantarum* B282. Furthermore, *L. plantarum* B282 failed to colonize the olive surface at 10% NaCl (Blana et al., 2014). Bella di Cerignola, inoculated with *L paracaseie* IMC2.1, showed a considerable genetic diversity (mainly at 8% NaCl) but *L. pentosus* was the most frequently isolated species. Hence, the results suggest that *L. pentosus* from autochthonous microflora might eventually have a favorable competence against strains from other species or environments.

## Conclusion

The predominance of potential probiotic strains during large-scale fermentations is not a risk-free challenging task due to the current conditions prevailing in the industrial fermentation yards, the competitiveness of the wild environmental microflora, and to the possible integration of *Enterobacteriaceae* into the polymicrobial biofilm formed on the olive surface. A detailed study of the physicochemical and microbiological changes during fermentation showed that: (i) an immediate post-brining inoculation (to reduce the presence of initial wild microorganisms), (ii) the use of a re-inoculation, to replace the LAB that could have eventually died after the first one, and (iii) an early processing in the season led to a general improvement of the inoculum survival. On the contrary, inoculum history (laboratory vs. industry pre-adaptation) or HCL addition (25 L vs. 10 HCl L/vessel) had not or limited influences. The complete inhibition of LAB2 (and almost any LAB) during the storage phase in the inoculation experiments conducted in September was unexpected and is currently under investigation.

## Author Contributions

FR-G, VR-G, JR-R, RT-G, and JB-G performed the experimental work. FR-G, AG-F, PG-G, and FA-L designed the work, analyzed the results and wrote the paper.

## Conflict of Interest Statement

The authors declare that the research was conducted in the absence of any commercial or financial relationships that could be construed as a potential conflict of interest. The reviewer CM and handling Editor declared their shared affiliation, and the handling Editor states that the process nevertheless met the standards of a fair and objective review.
