# Supplementary material for: Assessing the Challenges in the Application of Potential Probiotic Lactic Acid Bacteria in the Large-Scale Fermentation of Spanish-Style Table Olives
Source: Front Microbiol. 2017 May 17;8:915. doi: 10.3389/fmicb.2017.00915 (PMC5434132; doi:10.3389/fmicb.2017.00915)
Supplement: Supplementary file 1 [file Data_Sheet_1.docx]

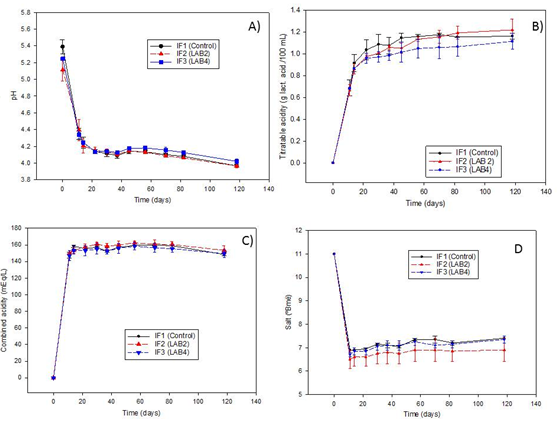


**Figure S1.** Tentative probiotic industrial fermentation (season 2011/2012). Changes in the values of pH (A), titratable acidity (B), combined acidity (C), and salt (D) during fermentation process. Correspondence between symbols and treatments: IF1, control, IF2, inoculation with LAB2; IF3, inoculation with LAB4.


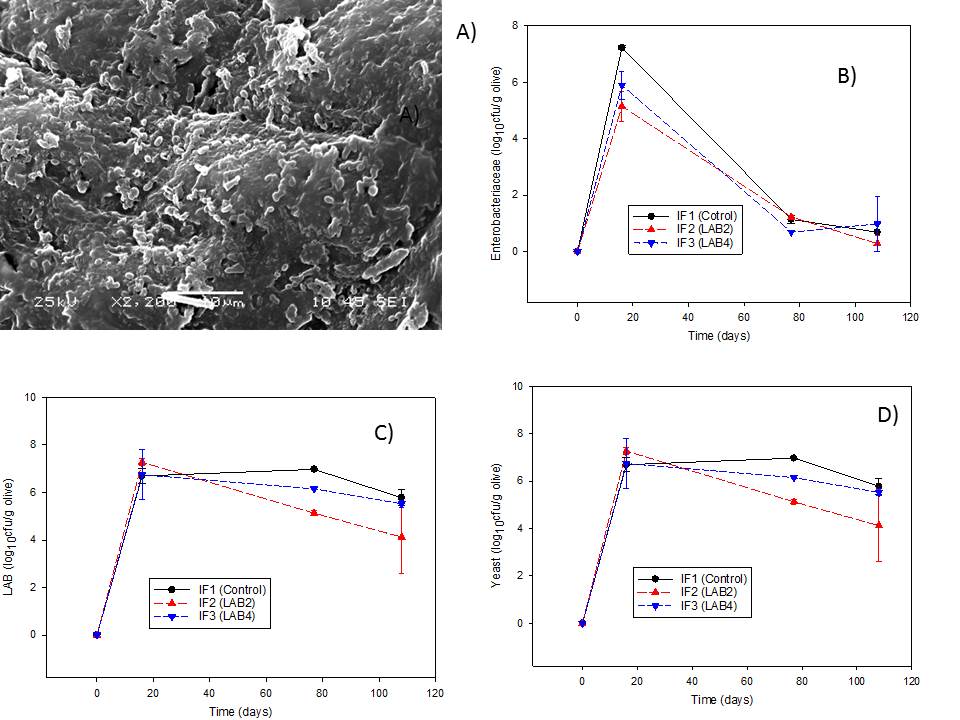


**Figure S2**. Tentative probiotic industrial fermentation (season 2011/2012). Example of biofilm formation on the olives (A) and changes in the *Enterobacteriaceae* (B), LAB (C), and yeast (D) loads on the polymicrobial biofilm on the olive surface. Correspondence between symbols and treatments: IF1, control, IF2, inoculation with LAB2; IF3, inoculation with LAB4.


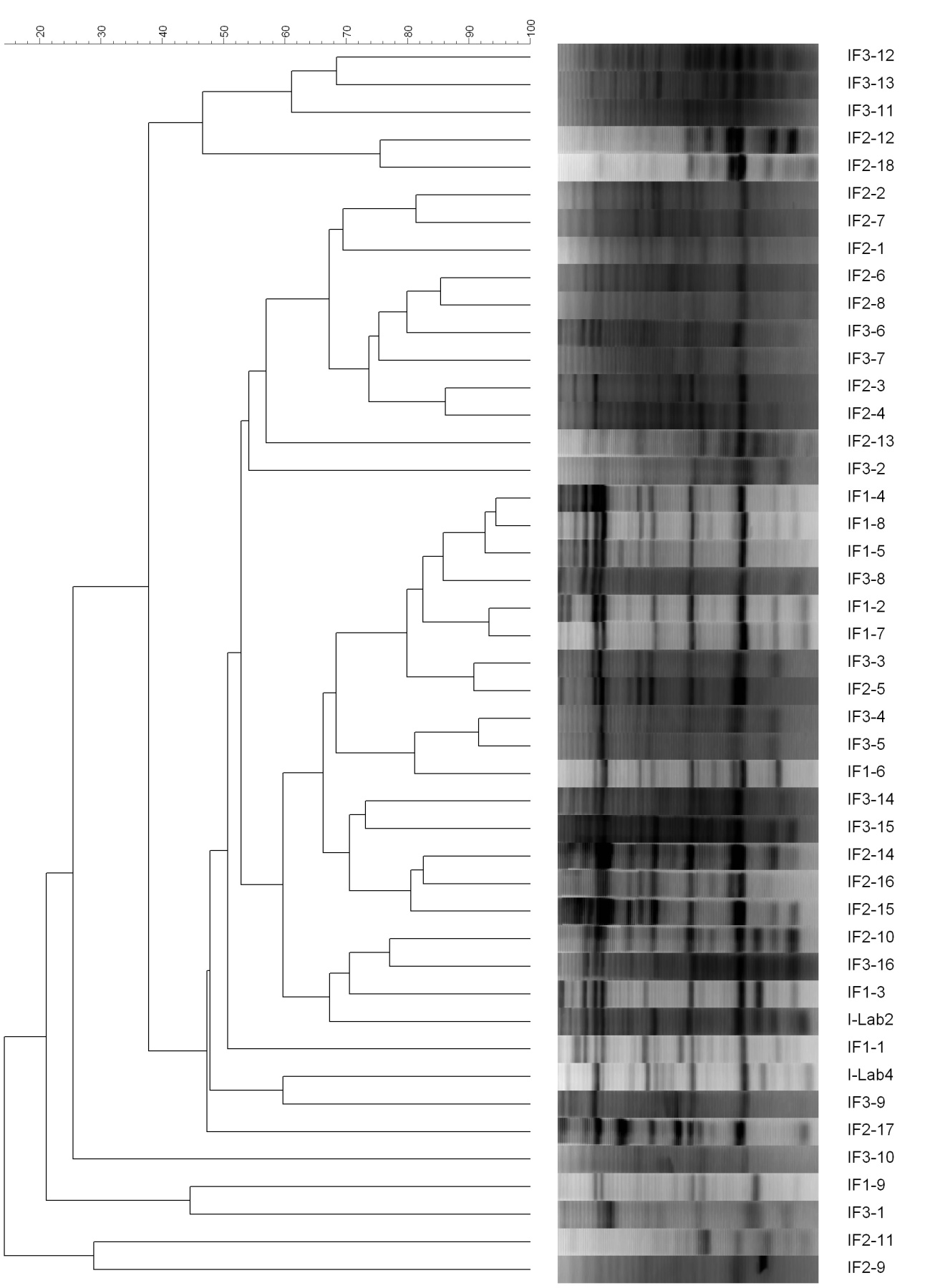


**Figure S3**. Tentative probiotic industrial fermentation (season 2011/2012). Dendrogram relative to the LAB strain isolates at the moment of maximum population.Correspondence between symbols and treatments: IF1, control, IF2, inoculation with LAB2; IF3, inoculation with LAB4.


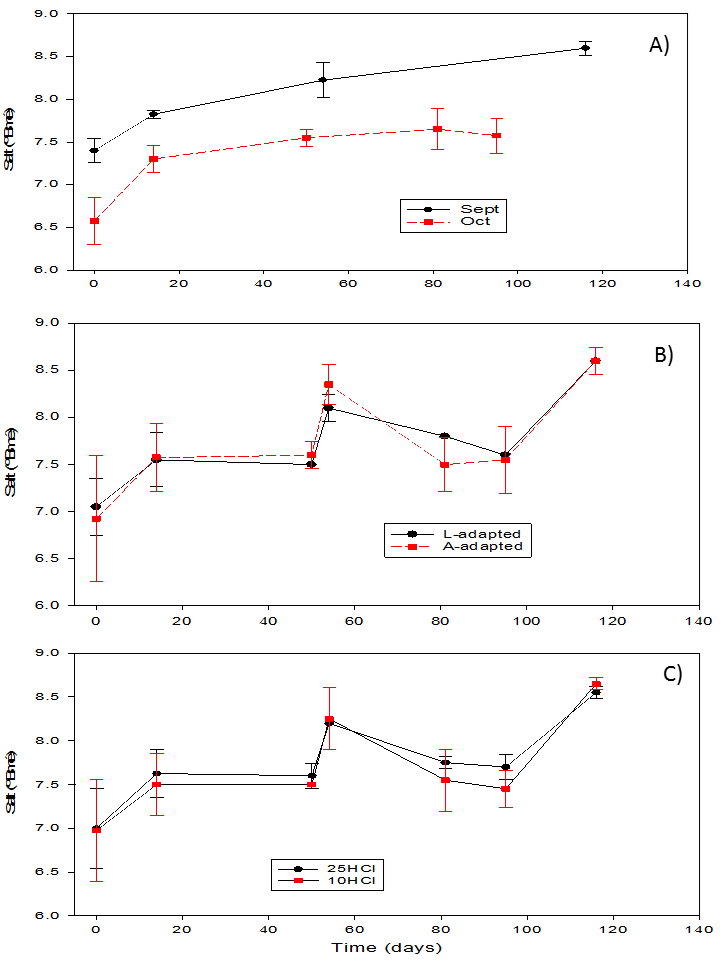


**Figure S4**. Application of potential probiotic starter (LAB2) at industrial scale (season 2012/2013). Changes in salt content (±CL) as a function of season time (upper panel), type of inoculum (middle panel), and proportion of food grade HCl added to fermentation vessels (bottom panel). The values are always average over the other variables non-included in the graph.


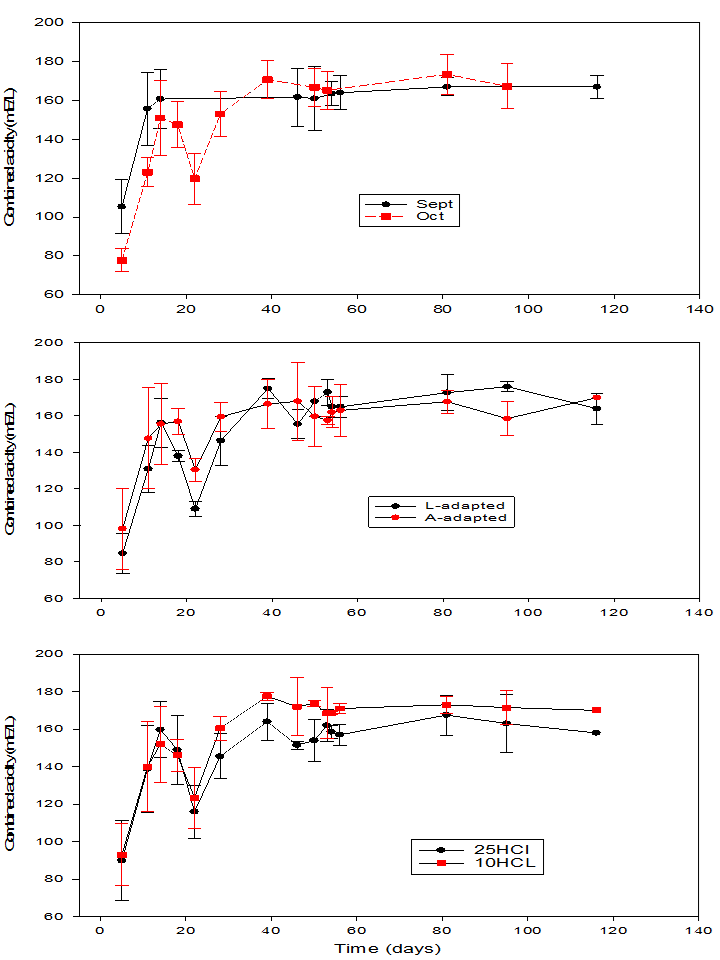


**Figure S5**. Application of potential probiotic starter (LAB2) at industrial scale (season 2012/2013). Changes in combined acidity (±CL) as a function of processing season time (upper panel), type of inoculum (middle panel), and proportion of food grade HCl added to fermentation vessels (bottom panel). The values are always average over the other variables non-included in the graph.


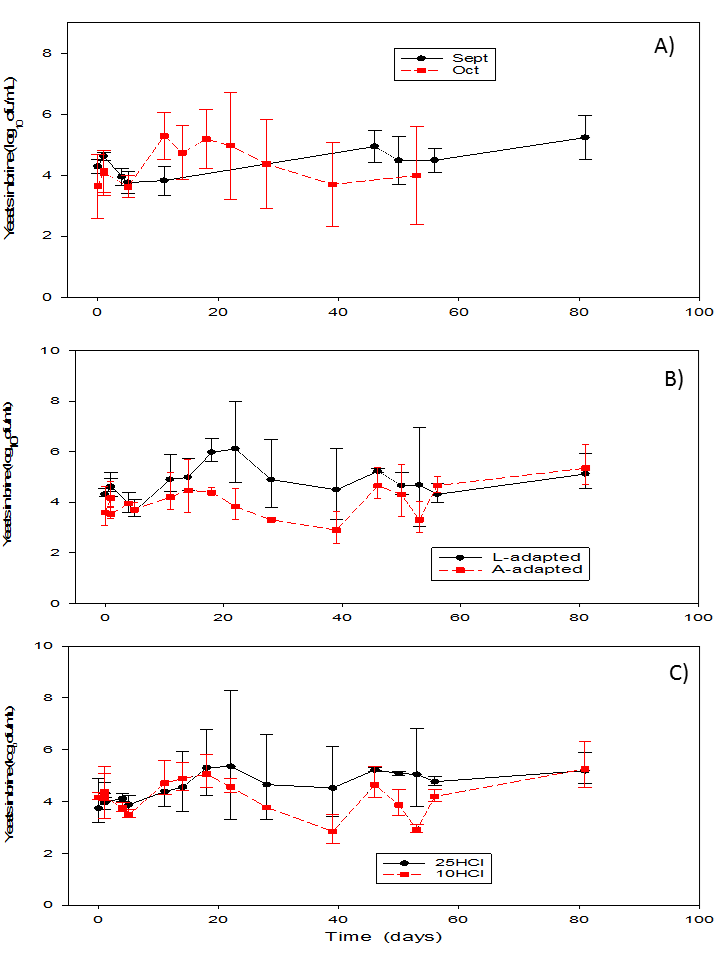


**Figure S6** Application of potential probiotic starter (LAB2) at industrial scale (season 2012/2013). Changes in yeast population in brine (±CL) as a function of processing season time (upper panel), type of inoculum (middle panel), and proportion of food grade HCl added to fermentation vessels (bottom panel). The values are always average over the other variables non-included in the graph.


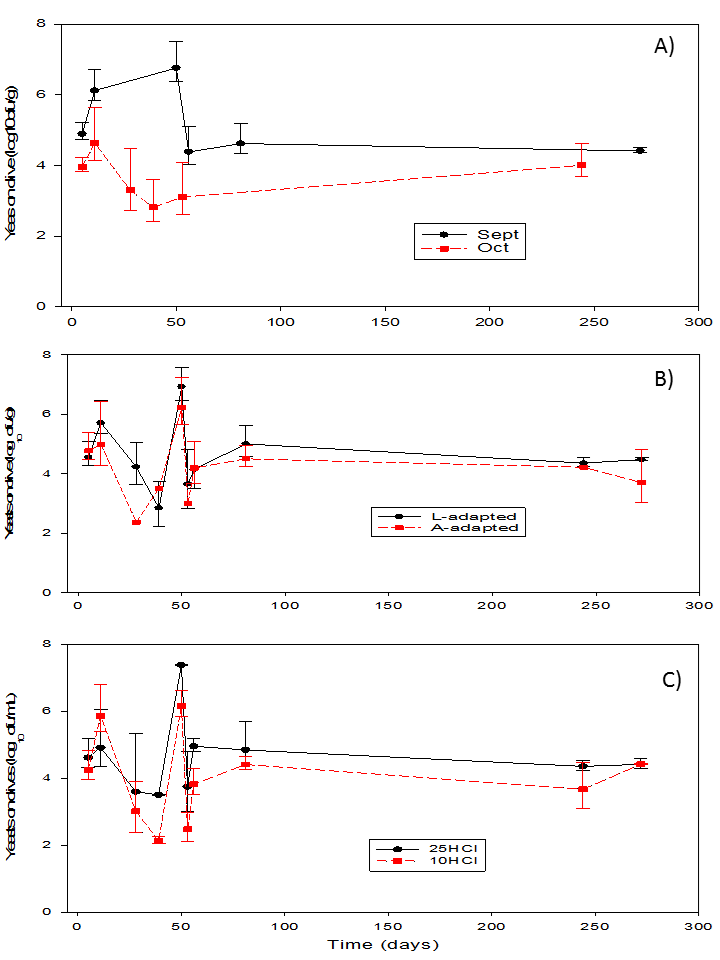


**Figure S7**. Application of potential probiotic starter (LAB2) at industrial scale (season 2012/2013). Changes in yeast population on olives (±CL) as a function of processing season time (upper panel), type of inoculum (middle panel), and proportion of food grade HCl added to fermentation vessels (bottom panel). The values are always average over the other variables non-included in the graph.
